# *In vivo* Evaluation of Single Dose Tetanus Toxoid Vaccine Formulation with Chitosan Microspheres

**DOI:** 10.4103/0250-474X.40325

**Published:** 2008

**Authors:** R. Manivannan, S. A. Dhanaraj, Y. Udaya Bhaskara Rao, A. Balasubramaniam, N. L. Gowrishankar, N. Jawahar, S. Jubie

**Affiliations:** J. S. S. Centre for Research and Post Graduate Studies, J. S. S. College of Pharmacy, Ootacamund - 643 001, India; 1 Swamy Vivekanandha College of Pharmacy, Elayampalayam (Thiruchengode) - 637 205, India; 2 Pasteur Institute of India, Coonoor - 643 103, India

**Keywords:** Tetanus toxoid, microspheres, chitosan, tetanus toxin, tetanus antitoxin

## Abstract

Chitosan adsorbed microspheres containing tetanus toxoid were prepared in the size range of 10 μm to 75 μm, by emulsion-cross linking technique at different speeds of agitation. The amount of tetanus toxoid incorporated into chitosan microspheres were estimated by limes flocculation test and *in vivo* evaluation of tetanus toxoid adsorbed chitosan microspheres were determined by toxin neutralization method using albino mice. The results of *in vivo* release for the batches of 10 μm and 25 μm correlates with the results of *in vitro* in which both the batches passes the limit of IP standard (4 Lf) where as, for the batches of 50 μm and 75 μm, the *in vitro* release of tetanus toxoid was 2 Lf. But our *in vivo* studies for the batches of 50 μm and 75 μm fail to pass the limit stated in IP. The release of tetanus toxoid from the chitosan microspheres was found to be sustained for the period of 6 months.

Controlled delivery of bioactive macromolecules has become increasingly important. Vaccines are one of the important classes of molecules that could potentially benefit from controlled release because many immunization schedules require administration of several spaced antigen doses. In particular the conversion of multiple-dose vaccines in to single-dose vaccines may represent an important advancement, which should lead to improved vaccination coverage, as well as to a reduction in vaccination costs^1^.

Many vaccines against pathogenic infections such as tetanus, diphtheria, pertussis, poliomyelitis and hepatitis B require more than one dose for complete immunization of a child. The multidose schedule of immunization increases the cost of immunization several fold, as well as having the disadvantages of inconvenience and incomplete subject compliance. To overcome these shortcomings, technologies have been developed to formulate single contact point delivery systems for vaccines requiring multiple injections^2^. This project is to develop a controlled release tetanus toxoid vaccine formulation, which could replace the two or three doses, required for primary immunization series.

The initial priority was to develop a single-dose tetanus vaccine, as one of the most urgent needs of the Expanded Programme on Immunization is to control neonatal tetanus. According to World Health Organization (1999) statistics, each year, more than 4 50 000 infants die from neonatal tetanus (tetanus in the first month of life), and nearly 40 000 mothers die from tetanus infection acquired during delivery. So, the prevention of neonatal tetanus would greatly benefit from the development of vaccine requiring only one injection in pregnant women or women childbearing age. However, this technology if successful, will also be applied to other vaccines, namely DPT and hepatitis B and possibly inactivated polio vaccine. For this work, tetanus toxoid has been selected because of its characteristics and availability and appears as a suitable antigen to begin alternative approaches to the existing vaccines.

Tetanus Toxoid is a protein product prepared from tetanus toxin from the growth of a highly toxicogenic strain of tetanus bacilli, *C. tetani* in a suitable medium. The concentrated and purified tetanus toxoid is adsorbed on a mineral adjuvant (aluminium hydroxide/aluminium phosphate) to enhance considerably its immunizing activity^3^.

Chitosan is a high molecular weight, cationic polysaccharide obtained by the deacetylation of chitin^4^, the major compound of exoskeletons of crustaceans. Because of its low production costs, biodegradability, biocompatibility, bioadhesivity, non-toxic^5^,^6^ in nature and recent FDA approval, the pharmaceutical and food applications of chitosan have increased remarkably over recent years.

## MATERIALS AND METHODS

Tetanus toxoid (700 L_f_/ml), tetanus toxin, tetanus antitoxin and tetanus antitoxin-national reference was obtained from Pasteur Institute of India, Coonoor. Chitosan was a gift sample from Central Institute of Fisheries Technology, Cochin. Linseed oil, toluene, glutaraldehyde (AR Grade), were purchased from S. D. Fine Chemicals Ltd, Mumbai. All the other chemicals used in the present study were of AR Grade. For conducting *in vivo* experiments, animals were procured from the central Animal House of the Institute. All animal experimental protocols were approved by the Institutional Animals Ethics Committee.

### Preparation of chitosan-gel microspheres^7^:

Chitosan-gel microspheres were prepared by emulsion-crosslinking technique in which glutaraldehyde was used as the cross-linking agent. The solutions of 1.5% chitosan and 2.0% chitosan were prepared in aqueous acetic acid (3%) containing sodium chloride (2%) and this was stirred at 3000 rpm to form a gel. A dispersion phase was prepared by mixing 50 ml of toluene and 5 ml of span-80 using stainless steel remistirrer at 1000 rpm speed for 10 min. To this 4 ml of chitosan gel with 1 ml of 0.01 N hydrochloric acid was added and the stirring was continued, then glutaraldehyde saturated toluene (5 ml) was added drop wise. Finally 4% glycine (2 ml) was added to cap any free aldehyde groups. The microspheres were collected and washed in acetone and toluene with centrifugation and they were dried at room temperature and stored in desiccator.

### Loading of tetanus toxoid:

The tetanus toxoid was incorporated into chitosan gel microspheres by adsorption method. In the preparation of tetanus toxoid loaded chitosan microspheres, 400 mg of the pooled 1.5% empty microspheres were mixed with 40 ml of sterile water and stored for 24 h, to achieve proper swelling. The swelled microspheres were separated by centrifugation at 7000 rpm then washed with acetone. These microspheres were immersed in about 4 ml of plain tetanus toxoid (700 L_f_/ml) and kept at 37° for 20 h adsorption. The microspheres were centrifuged and unadsorbed supernatant tetanus toxoid (0.75 ml) was removed. Then the tetanus toxoid adsorbed 1.5% chitosan microspheres were washed with methanol and acetone and they were taken for further evaluation^8^. Three other batches of microspheres were prepared by the above mentioned method with 2% chitosan. The Table 1 shows the different batches of tetanus toxoid adsorbed chitosan microspheres were used for *in vivo* studies.

**TABLE 1:** DIFFERENT BATCHES OF FORMULATION WERE USED FOR *IN VIVO* STUDIES

Different batches of formulation
Tetanus toxoid adsorbed onto 1.5% chitosan microspheres (10 μm)
Tetanus toxoid adsorbed onto 1.5% chitosan microspheres (25 μm)
Tetanus toxoid adsorbed onto 2.0% chitosan microspheres (50 μm)
Tetanus toxoid adsorbed onto 2.0% chitosan microspheres (75 μm)
Mixed batches (1.5% and 2.0%)
Pasteur Institute Vaccine (Control)

Six different batches of formulation using 1.5% (10 μm and 25 μm) and 2.0% (50 μm and 75 μm) Chitosan microspheres were used for *in vivo* studies

The amount of tetanus toxoid incorporated into chitosan microspheres were estimated by limes flocculation (L_f_) test. Limes flocculation means, when the concentration of toxin, or toxoid, is kept constant and the concentration of the antitoxin varied in mixtures of constant volume, the mixture flocculating first is that which contains the most nearly equivalent quantities of toxin, or toxoid and antitoxin^9^.

### *In vivo* evaluation of microspheres:

The suggested method in the Indian Pharmacopoeia is to determine the potency of tetanus vaccine is antibody induction method (toxin neutralization method). The various steps are involved in the antibody induction method, immunization of guinea pigs, determination of Lp/10 dose, collection of immune sera from guinea pigs, titration of standard antitoxin (control titration) and titration of immune guinea pig sera at Lp/10 level.

### Immunization of guinea pigs^10^:

The guinea pigs for test and control group were selected. The animal ranged from weights 250-350 g and quarantined for 1 w before use with regular diet. The tetanus toxoid adsorbed chitosan microspheres were dispersed in aqueous vehicle (0.5% sorbitol, 0.1% carboxymethylcellulose and 0.002% Tween-80) to get a final concentration of 0.5 Lf/ml. These six samples were injected subcutaneously into six groups of guinea pigs.

### Collection of immune sera from guinea pigs^11^:

Bleeding of the immunized guinea pigs were done by cardiac puncture under general anaesthesia (with anesthetic ether) using 20 gauze needle. The blood samples were collected in sterile glass tubes and kept in a slanting position to allow the serum to ooze out of the clotted blood. Then these tubes containing clotted blood and serum were kept at 37° for 1 h and stored at 4° in for 24 h. The serum was separated by centrifugation at 2500 rpm for 15 min and the serum of each guinea pig accordingly labeled and stored at −20° before performing neutralization test.

### Determination of *Limes paralyticum*/10 (Lp/10) dose of the test toxin:

The *Limes paralyticum*/10 dose of the test toxin is the smallest quantity of the toxin, which mixed with 0.1 unit of the standard preparation and injected subcutaneously into mice causes tetanic paralysis within 4 d. For the determination Lp/10 dose, the mixtures were prepared by adding 2.0 ml of diluted solution of the tetanus antitoxin-national reference preparation into each of five graded volumes of diluted test toxin correspondingly to 2.32, 2.40, 2.48, 2.56, and 2.64 ml. Their volumes were made up to 5.0 ml with peptone water, so that 0.5 ml of each mixture contained 0.1 unit of tetanus antitoxin. Mixtures were incubated at room temperature protected from 15-60 min. After incubation, the mice were inoculated with 0.5 ml of mixture per mouse and the injected mice were observed for 4 d.

### Titration of standard antitoxin (control titration):

Control titration was carried out along with the neutralization test to check the validity of the Lp/10 test. From the solution of diluted standard antitoxin, five different concentrations were prepared by adding correspondingly 1.6, 1.8, 2.0, 2.2 and 2.4 ml of diluted standard antitoxin. To this 2.0 ml of diluted toxin was added and the volume was made upto 5.0 ml with peptone water, so that 0.5 ml of each mixture contained 0.0060 ml of toxin. The mixtures were incubated at room temperature, protected from light for 15-60 min and then inoculated into each mouse.

### Titration of immune guinea pig sera at Lp/10 level:

The neutralization test was carried out on pooled sera to obtain the end point (the international unit per ml), the pooled sera was diluted further accordingly such that the end point value was determined forunitage in different intervals of bleeding.

## RESULTS AND DISCUSSION

From our studies, it was observed that the size of microspheres increased with the decrease in the speed of agitation. The amount of drug loaded was 70-84% by limes flocculation test. The biodegradable microspheres made up of chitosan polymer having good spherical geometry, adsorbed onto tetanus toxoid (plain) was used for protracted pulsed release of the incorporated toxoid to reduce multiple immunization regimes. The size of the microspheres was found to be ranging between 10-75 μm.

From our studies, 0.006 ml (Table 2) of standard toxin dose per mouse was the smallest quantity of the toxin which when mixed with 0.1 unit of the standard antitoxin preparation caused tetanic paralysis with in 4 d and this was used as the Lp/10 dose. After ascertaining the level of Lp/10 value of tetanus toxin, the standard titration was done using the same quantity of tetanus toxin (0.006 ml) and different quantities of standard antitoxin (0.1 IU/ml). The Table 3 shows the tetanus toxoid adsorbed onto different size (10 μm, 25 μm, 50 μm, 75 μm and mixed batches) of chitosan microspheres have elicited specific antibodies at different level.

**TABLE 2:** DETERMINATION OF Lp/10 (LIMES PARALYTICUM/10) DOSE OF THE TEST TOXIN

Toxin dose (ml)	Antitoxin dose (TNR) IU	Mixtures table				Observation			
	
		Dil.toxin (ml)	Dil.antitoxin (ml)	Peptone water (ml)	Total volume (ml)	1^st^ day	2^nd^ day	3^rd^ day	4^th^ day

0.0058	0.1	2.32	2.0	0.68	5.0	√√√	√√√	√√√	ttttt,
						√√√	√√√	√√√	tttt
0.006	0.1	2.40	2.0	0.60	5.0	√√√	√^tt^	√^tt^	ttttttt,
						√√√	√√√	√√√	ttttttt
0.0062	0.1	2.48	2.0	0.52	5.0	√√√	tttt	tttt	DDD
						√√√	ttttttt	ttttttt	
0.0064	0.1	2.56	2.0	0.44	5.0	√√√	ttttttttt	ttttttttt	
						√√^t^	tttttt^tt^	tttttt^tt^	
0.0066	0.1	2.64	2.0	0.36	5.0	√√√	Dtttttt	Dtttttt	
						√^tt^	ttttttttt	ttttttttt	

√ -Normal mouse

t - tetanic paralysis of one limb

tt - tetanic paralysis of two limb

ttt - tetanic paralysis of three limb

D - death due to tetanus

**TABLE 3:** MAXIMUM POTENCY UNITAGE ESTIMATED FOR DIFFERENT VACCINE FORMULATIONS AT DIFFERENT LEVEL

Different vaccine formulations	Unitage (lU/ml)			
	
	2^nd^ week after booster dose	6^th^ week after booster dose	9^th^ week after booster dose	12^th^ week after booster dose

1.5% TT adsorbed chitosan batch (10 μm)	>0.5	2.0	2.0	1.0
1.5% TT adsorbed chitosan batch (25 μm)	>0.5	2.0	2.0	1.5
2.0% TT adsorbed chitosan batch (50 μm)	>0.5	0.5	<0.5	<0.5
2.0% TT adsorbed chitosan batch (75 μm)	>0.5	0.5	<0.5	<0.5
Mixed batches (10 + 25 + 50 + 75 μm)	>0.5	1.0	1.0	1.0
Control batch (PIIC)	>0.5	2.0	4.0	4.0

TT is tetanus toxoid, PIIC - Pasteur Institute of India, Coonoor

The 10 μm microspheres of 1.5% chitosan have elicited a specific antibody level of 0.5 IU/ml to 2.0 IU/ml in the sera for 2^nd^ and 6^th^ w after the booster dose, respectively. The antibody level of 2.0 IU/ml remained the same in 9^th^ w serum sample. Although the immune response elicited by 1.5% tetanus toxoid adsorbed chitosan (10 μm) batch has decreased to 1.0 IU/ml after the 12^th^ w of booster dose, but it has passed the Indian Pharmacopoeia standard^9^.

The 25 μm microspheres of 1.5% chitosan also elicited the same response as 10 μm microspheres, but the antibody level after 12 w was did not go below 1.5 IU/ml as in the case of 10 μm batch. The 50 μm and 75 μm of 2.0% chitosan microspheres exhibited comparatively a lower antibody response, having the maximum antibody level of 0.5 IU/ml after 6 w. For these microsphere batches after 12 w the antibody level was reduced below 0.5 IU/ml. This may be due to the delay or obstructed release of antigen from the microsphere matrix.

The mixed batches (1.5% and 2.0%) containing all four different sizes of microspheres adsorbed with tetanus toxoid have elicited a lower antibody response when compared to the individual batches (10 μm and 25 μm). In this batch, the antibody response remained constant at 1 IU/ml after 6 w.

From these observations it was ascertained that 10 μm, 25 μm, and mixed batches of chitosan adsorbed were able to produce the antibodies to the required level mentioned in official monograph, but when the antibody level produced was compared with Pasteur Institute Vaccine (control) the antibody level was 50% less. Since for Pasteur Institute Vaccine (control), the antibody level was 2-4 IU/ml. From these results indicated that chitosan microspheres are capable of inducing required antibody titre, it is necessary to study the factors which can improve the microspheres system for eliciting the antibody level equivalent to Pasteur Institute Vaccine (control) and also for a longer antibody induction period.

The present study suggested that the maintenance of antibody level has to be estimated over a period of 6 mo so as to confirm the pulsatile releasing mechanism of chitosan microspheres for the enhancement of immunogenicity and for proving the efficiency of vaccine delivery system.
